# Microbial life on a sand grain: from bulk sediment to single grains

**DOI:** 10.1038/ismej.2017.197

**Published:** 2017-12-01

**Authors:** David Probandt, Thilo Eickhorst, Andreas Ellrott, Rudolf Amann, Katrin Knittel

**Affiliations:** 1 Max Planck Institute for Marine Microbiology, Department of Molecular Ecology, Bremen, Germany; 2 University of Bremen, Faculty 2 (Biology/Chemistry), Bremen, Germany

## Abstract

Globally, marine surface sediments constitute a habitat for estimated 1.7 × 10^28^ prokaryotes. For benthic microbial community analysis, usually, several grams of sediment are processed. In this study, we made the step from bulk sediments to single sand grains to address the microbial community directly in its micro-habitat: the individual bacterial diversity on 17 sand grains was analyzed by 16S ribosomal RNA gene sequencing and visualized on sand grains using catalyzed reporter deposition fluorescence *in situ* hybridization. In all, 10^4^–10^5^ cells were present on grains from 202 to 635 μm diameter. Colonization was patchy, with exposed areas largely devoid of any epi-growth (mean cell–cell distance 4.5±5.9 μm) and protected areas more densely populated (0.5±0.7 μm). Mean cell–cell distances were 100-fold shorter compared with the water column. In general, growth occurred in monolayers. Each sand grain harbors a highly diverse bacterial community as shown by several thousand species-level operational taxonomic units (OTU)_0.97_. Only 4–8 single grains are needed to cover 50% of OTU_0.97_ richness found in bulk sediment. Although bacterial communities differed between sand grains, a core community accounting for >50% of all cells was present on each sand grain. The communities between sediment grains are more similar than between soil macroaggregates.

## Introduction

The top 10 cm of marine sediments constitute a habitat for estimated 1.7 × 10^28^ *Bacteria* and *Archaea* (Whitman *et al.*, 1998). In surface sediments, cell abundances are 10^8^–10^9^ cm^−3^ (Dale, 1974; Meyer-Reil *et al.*, 1978; Llobet-Brossa *et al.*, 1998; Musat *et al.*, 2006) making the benthic microbial community up to 10 000 times more dense than the one in the water column. In sandy sediments, >99% of the benthic microbial community lives attached to sand grains (Rusch *et al.*, 2003). High mixing rates because of shallow water depths and strong currents resuspend sand grains and expose its microbial community to mechanical shearing stress (Miller, 1989) and highly dynamic environmental conditions (Huettel *et al.*, 2014). The sediment filters and accumulates organic and inorganic matter from the environment. However, the availability of the organic and inorganic matter and oxygen changes regularly with bottom water current-induced bedform migration (Ahmerkamp *et al.*, 2017). Overall, sands are spatiotemporal heterogeneous microbial habitats that provide manifold ecological niches to benthic microbial communities. Some microbes produce extracellular polymeric substances to allow an attachment to sand grains’ surfaces (Flemming and Wingender, 2010) and the establishment of a diverse microbial community.

Benthic microbial communities are metabolically diverse, which is reflected in their phylogenetic composition. For North Sea surface sediments, bacterial operational taxonomic unit (OTU)_0.97_ richness was between 3000 and 12 000 as assessed by 16S ribosomal RNA (rRNA) gene sequencing of bulk sediment (Probandt *et al.*, 2017). Our current knowledge about the diversity, spatial organization and cell–cell interactions within benthic microbial communities is based on the analysis of bulk sediments and is, thus, limited in spatial resolution. The only studies investigating the spatial arrangement of benthic microbial communities on sand grains on a micro-scale were conducted decades ago and based on autofluorescence and morphology of microorganisms. Microalgae, *Cyanobacteria* and other bacteria live predominantly in protected areas (Wood and Oppenheimer, 1962; Meadows and Anderson, 1968; Weise and Rheinheimer, 1978; Miller, 1989). Reported cell densities on sand grains were 1 to 6 cells in 100 × 100 μm (DeFlaun and Mayer, 1983).

Making use of the major advances in microscopy that have become available in recent years (mainly detector sensitivity and brighter dyes), we went beyond the bulk sediment by taking a direct look at single sand grains (SSGs) to study the microbial community in its natural habitat. We established a workflow for (i) bacterial diversity analysis of the sand grain’s community using tag sequencing of partial 16S rRNA genes amplified from individual SSGs, and (ii) the direct visualization of microbial communities on native sand grains using catalyzed reporter deposition fluorescence *in situ* hybridization (CARD-FISH).

There are several factors influencing the (microbial) colonization of sand grains including neutral, taxa-independent factors, such as physical disruption (for example, Miller, 1989; Coyte *et al.*, 2017) or colonization probability, and deterministic, taxa-dependent factors, such as different adhesion capability (reviewed in Dang and Lovell, 2016) and growth behavior. Considering the high bacterial diversity in bulk surface sediments, which is represented by several thousand species (Newton *et al.*, 2013; Liu *et al.*, 2015; Probandt *et al.*, 2017), the low colonized fraction on a sand grain (4–30%, Meadows and Anderson, 1968), as well as colonizer effects, we hypothesize that the diversity and community composition would differ more strongly between sand grains than between replicates of the bulk sediment.

## Materials and methods

### Sampling

Samples were retrieved from subtidal sediments in the southern North Sea at site Helgoland Roads on 14 June 2016 (Supplementary Figure S1A). Sediment push cores were retrieved by scientific divers from a water depth of 8 m. Samples were transported to the lab and immediately sectioned. For DNA extraction and PCR, samples were stored at −20 °C. For CARD-FISH, SYBR green I and Acridine Orange staining, surface sediment (0–2 cm) was fixed with 1.5% formaldehyde for 1 h at room temperature, washed in 1 × phosphate-buffered saline/ethanol (1:1, v/v) and stored at −20 °C until use.

### Micro-computed tomography (μCT)

For μCT, we subsampled the center of undisturbed push cores with a polyethylene cylinder (14 mm diameter × 30 mm height). After dehydration in acetone, samples were impregnated with polyester resin (Eickhorst and Tippkötter, 2008). After polymerization, samples were visualized by X-ray μ-CT (CT-ALPHA system, ProCon, Sarstedt, Germany). For details, see Supplementary Information.

### DNA extraction from bulk sediments

A total of six independent DNA extractions from each 0.4 g (*ca* 8000 sand grains) of sediment (0–2 cm depth) were done. Three extractions were performed using the PowerSoil DNA isolation kit (MoBio, Carlsbad, CA, USA) and three extractions were performed according to Zhou *et al.* (1996) ending with two ethanol washing steps.

### Amplification of partial 16S rRNA genes

For PCR amplification of 16S rRNA gene fragments from bulk sediments, three DNA pools, each a mixture of an equal molar ratio from the two extraction methods, were used as a template. For each pool, five replicate PCR reactions (50 μl volume) were performed containing 0.3 mg μl^−1^ bovine serum albumin, 1 × TaKaRa buffer, 0.2 mM dNTPs, 1.5 μM of each primer S-DBact-0341-b-S-17 and S-D-Bact-0785-a-A-21 (Herlemann *et al.*, 2011; Klindworth *et al.*, 2013), 0.25 U μl^−1^ TaKaRa Ex Taq DNA Polymerase (TaKaRa Bio Inc., Kusatsu, Japan) and *ca* 20 ng DNA. The PCR program started with an initial denaturation step for 5 min at 95 °C, followed by 40 cycles of 95 °C for 60 s, 55 °C for 60 s, 72 °C for 180 s and a final extension step for 10 min at 72 °C.

In parallel, SSGs from the 0 to 2 cm depth interval were used as template for PCR without prior DNA extraction. Grains were randomly picked with sterile, DNA-free forceps, size and appearance documented by photographs and transferred into PCR strips (one grain per reaction) filled with sterile water. The protocol for SSG-PCR was as described for bulk sediments. For each grain, new forceps were used and dipped into a separate PCR reaction mix as a negative control before picking the grain. There was no PCR product in any of the negative controls. PCR products were excised from the agarose gel and sequenced on an Illumina (San Diego, CA, USA) platform (HiSeq2500, 2x250 bases, paired-end) at the Max Planck-Genome Center in Cologne (Germany).

### Quality trimming and sequence processing

Paired-end reads were quality trimmed (>q21, both ends) and merged (strict, overlap 20) using software package BBmap v36.92 (BBTools package, Brian Bushnell, Walnut Creek, CA, USA). Further read processing was done according to the MiSeq SOP (Kozich *et al.*, 2013) with mothur v.1.39.5 (Schloss *et al.*, 2009; Westcott and Schloss, 2017). Sequences were classified using the SILVA database SSU Ref NR, release 123 (Quast *et al.*, 2013) and globally clustered in OTUs at 97% sequence similarity. Rare OTU_0.97_ that were represented by <3 sequences in the whole data set, that is, single sequence OTU absolute (SSO_abs_), and double sequence OTU absolute (DSO_abs_), were removed prior to diversity analysis. For details, see Supplementary Information. Raw sequence data have been stored in the European Nucleotide Archive (ENA) under study accession number PRJEB20733.

### Diversity analysis

The alpha diversity was studied by phylotype-based Chao1 (Chao, 1984) and inverse Simpson (Simpson, 1949), as well as the phylogenetic measure Faith’s PD (Faith, 1992). The beta diversity was studied by phylotype-based comparative OTU_0.97_ presence/absence and phylogenetic measure of weighted and unweighted UniFrac (Lozupone and Knight, 2005). For analysis, R packages vegan (Oksanen *et al.*, 2013), ape (Paradis *et al.*, 2004), picante (Kembel *et al.*, 2010) and GUniFrac (Chen, 2012), as well as FastTree2 (Price *et al.*, 2010) were used on data sets subsampled to the lowest number of sequences in any sample. For details, see Supplementary Information.

### Total cell counts

Cells were dislodged from sand grains by ultrasonication (6 × 30 s at 20% and 2 × 30 s at 50% power; HD70 probe, Bandelin, Berlin, Germany). Supernatants were collected after each round of sonication and replaced by phosphate-buffered saline/ethanol. Afterward, cells were filtered on polycarbonate membrane filters (0.2 μm pore size; three technical triplicates), stained with Acridine Orange (Meyer-Reil *et al.*, 1978) and counted under an epifluorescence microscope (50i, Nikon Instruments, Düsseldorf, Germany).

### Glass slides for microscopy of sand grains

Standard glass slides were customized for visualization of microbial communities on sand grains (Supplementary Figure S2). Using a diamond drill (diameter 10 mm), a hole was carefully drilled into the glass slide. A coverslip was attached to the slide using double-sided self-adhesive sticky frames (Gene Frame AB-0577, Thermo Fisher Scientific, Waltham, MA, USA). Dried sand grains were placed on the coverslip.

### SYBR green I staining

Cells on sand grains were directly stained on the custom-made glass slide, by embedding the grains in Mowiol 4-88 (pH 7.5, adjusted with ascorbic acid) containing SYBR green I (final concentration 25 ×).

### Catalyzed reporter deposition fluorescence *in situ* hybridization (CARD-FISH)

All steps of the CARD-FISH protocol were applied to about 100–500 sand grains in 2 ml reaction vials. Permeabilization of cell walls was done at 37 °C with lysozyme (10 mg ml^−1^) for 60 min followed by achromopeptidase treatment (60 U ml^−1^) for 30 min. Endogenous peroxidases were inactivated in 0.01 m HCl containing 0.15% H_2_O_2_ for 20 min. Hybridization (4 h) using horseradish peroxidase-labeled probes and CARD step (1 h) were performed as described previously (Pernthaler *et al.*, 2002). During hybridization, vials were carefully inverted every 30 min to allow an efficient mixing of the hybridization buffer and sand grains. After each step, sand grains were equilibrated by replacing the supernatant at least three times with the solution needed for the following step.

For multiple hybridizations, horseradish peroxidase from the first probe was inactivated in 0.01 m HCl with 0.15% H_2_O_2_ for 20 min. Tyramides (1.4 μg ml^−1^) were labeled with Alexa488, Alexa594 or Alexa647. For visualization of a fourth population, Alexa594- and Alexa488-labeled tyramides were added in an equimolar ratio to the amplification buffer. For microscopy, sand grains were placed on the customized glass slide and embedded in Citifluor/Vectashield (4:1) containing 0.5 μg ml^−1^ 4′,6-diamidino-2-phenylindole (DAPI). Probes and formamide concentrations used are given in Supplementary Table S1.

### Probe design

Probes NM478 and NM645 targeting *Nitrosomonas* and *Nitrosospira*-related organisms were developed with the probe design tool implemented in ARB (Ludwig *et al.*, 2004) based on sequences from this study and SILVA database SSU Ref NR, release 123 using the tree provided in release 128. The probes were tested at varying formamide concentrations from 10 to 60% at 46 °C hybridization temperature. The highest possible formamide concentration at which signals were still bright enough for detection was selected for subsequent hybridizations.

### Image acquisition using inverse confocal laser scanning microscopy and cell–cell distance measurements

Visualization of microbial communities on sand grains was done by inverse laser scanning microscopy (LSM780, Zeiss, Jena, Germany). DAPI, SYBR green I, Alexa488, Alexa594 and Alexa647 were excited using lasers of 405, 488 , 488, 561 and 633 nm wavelength, respectively. Non-confocal images of sand grain surfaces were obtained by transmission light microscopy in bright field mode (aperture diaphragm entirely opened). For three-dimensional visualization of cells on surfaces, images of z-stacks were optimized by deconvolution using AutoQuant (Media Cybernetics, Rockville, MD, USA) and further processed using IMARIS (Bitplane, Zurich, Switzerland).

Mean cell–cell distances on sand grains were measured on maximum intensity projection images based on z-stacks (IMARIS). Automated cell detection and distance measurement were performed with the software ACMEtool 3 (July 2014; M Zeder, Technobiology GmbH, Buchrain, Switzerland; see also Supplementary Information and Figure S3).

### Calculations of cell density, colonized surface area and cells per sand grain

The colonization density (cells μm^−2^) was calculated based on total cell counts cm^−3^ (determined on membrane filters; 1.1±0.3 × 10^9^ cells cm^−3^) and grain surface area (1.2 × 10^10^ μm^2^ cm^−3^ sediment) determined by μCT imaging (for detail, see Supplementary Information).

## Results and Discussion

### Microbial colonization density on sand grains

The microbial colonization density on sand grains was calculated based on the grain’s surface area as analyzed by μCT imaging (Supplementary Figures S1B and C) and total cell counts as detected by Acridine Orange staining. Microbial cell numbers in surface sediments (0–2 cm depth) from site Helgoland Roads were 1.1±0.3 × 10^9^ cm^−3^ and thereby in the upper range as reported for other sandy sediments (Dale, 1974; Meyer-Reil *et al.*, 1978; Llobet-Brossa *et al.*, 1998; Rusch *et al.*, 2003). The colonization density was 0.09 cells μm^−2^ corresponding to one cell in an area of 11.1 μm^2^ and a theoretical average distance between two cells of 3.3 μm. This colonization density is about one to two orders of magnitude higher than values, which based on nitrogen sorption reported by Ahmerkamp (2016) for similar North Sea sediments and by DeFlaun and Mayer (1983) for an intertidal mudflat. Owing to the limited resolution of μCT (voxel edge length: 6.2 μm) total surface area was likely underestimated resulting in an overestimation of colonization density. In contrast, nitrogen sorption usually results in an underestimation of colonization density because of an overestimation of surfaces because of the inclusion of nanometer-sized, non-inhabitable pores into the analysis (DeFlaun and Mayer, 1983). The substantial fraction of nanometer pores in intertidal mudflats that are rich in clay and silt, explains the low colonization density.

Based on the footprint of 0.43 μm^2^ for an average cell and the colonization density of 0.09 cells μm^−2^, 4% of the grain’s’ surface is colonized. This supports previous estimations based on Ziehl–Neelsen staining by Meadows and Anderson (1968) who found a surface fraction of 4–30% to be colonized. Each sand grain is populated by 1.2 × 10^4^–1.1 × 10^5^ cells (according to Eq. I; grain size 202–635 μm). Similar numbers are obtained when dividing total cell numbers cm^−3^ by number of sand grains cm^−3^ (according to Eqs. II and III), which resulted in 8.2 × 10^3^–2.6 × 10^5^ cells per grain.

### Visualization of microbial populations on sand grains

The major aim of this study was to go beyond the bulk sediment level. For this purpose, individual sand grains were embedded with mounting medium containing SYRB green I and placed on a customized glass slide for inverse laser scanning microscopy. Several decades ago, microbial colonization of sand grains was documented by sketches of light microscopy observations (Meadows and Anderson, 1968) or studied by autofluorescence of chlorophyll (Wood and Oppenheimer, 1962). Later, scanning electron microscopy (Weise and Rheinheimer, 1978; Kenzaka *et al.*, 2005; D'Onofrio *et al.*, 2010) and epifluorescence microscopy (DeFlaun and Mayer, 1983) was used to study microbial life and physical interactions of cells on sand grains. SYBR green I staining in combination with inverse confocal laser scanning microscopy performed in this study, however, provides three other advantages over scanning electron microscopy despite a lower resolution: (i) it does not require a long or complex sample preparation, (ii) it allows the differentiation between cells and organic debris and (iii) it enables images of whole sand grains.

**Figure 1:**
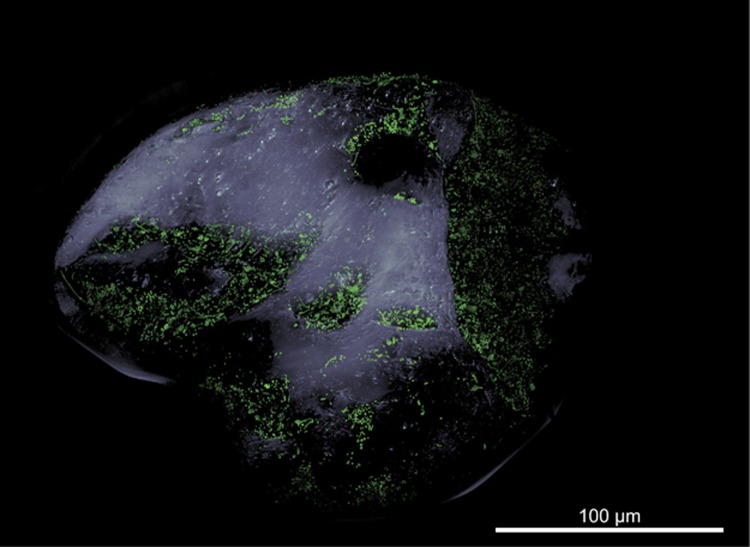
Microbial colonization of a sand grain. Confocal laser scanning micrograph showing SYBR green I-stained microbial cells on a sand grain visualized as three-dimensional reconstruction. The grain’s surface was visualized by transmitted light microscopy. Note the bare surfaces of convex and exposed areas in contrast to protected areas dominated by macrotopography, which are densely populated by microbes.

In general, all common microbial morphotypes such as cocci, short and long rods, as well as filaments could be visualized on the sand grain indicating a morphologically diverse community (Figure 1, Supplementary Figure S4C). Detected cells did not grow in multiple layers but rather grew as a monolayer. We defined two types of areas on the sand grains based on their surface topography: (i) exposed areas characterized by microtopography and mainly convex and smooth surfaces and (ii) protected areas with micro- and macrotopography. Cell–cell distances in exposed areas were between 0 and 29 μm (mean 4.5±5.9 μm), whereas mean cell–cell distances in protected areas were about 10-fold shorter (mean 0.5±0.7 μm, 0–14 μm; Supplementary Figure S4). As the ACME tool used for cell identification could not separate touching cells, mean cell–cell distances are based on non-touching cells only and therefore are overestimated. These measured cell–cell distances supported the calculated value of 3.3 μm. Similar to previous reports (Meadows and Anderson, 1968; Weise and Rheinheimer, 1978; DeFlaun and Mayer, 1983), microbial growth was preferentially found in well-protected areas of the sand grains. In contrast, exposed surfaces were largely unpopulated. Mechanical abrasion because of frequent and strong sediment transport processes (Meadows and Anderson, 1968; Miller, 1989) and strong pore-water advection (Precht and Huettel, 2004), both typical processes in surface sediments, are likely responsible. In laboratory settings, exposed and convex surfaces in undisturbed sediments can be readily colonized (Meadows and Anderson, 1968; Miller, 1989) as it has also been observed for surfaces of slow sand filters (Joubert and Pillay, 2008). Observed bare surfaces may, therefore, indicate a dynamic regular reworking of studied surface sediments preventing the development of bacterial growth on exposed areas. Another cause for bare surfaces may be grazing by large eukaryotic predators, which may not reach into well-protected areas of small indents.

### Bacterial diversity on SSGs versus diversity in bulk sediment

The bacterial diversity and community composition on individual sand grains was studied by Illumina tag sequencing of the V3-V4 region of 16S rRNA genes. For individual PCRs, we used a SSG as template. In total, 17 SSG-PCR products (of 22 SSG-PCRs) were obtained for sequencing. After rigid quality trimming, we obtained between 44 901 and 58 769 sequences for each of the 17 sand grains.

Each grain harbored a tremendous bacterial diversity as shown by 3426–6031 observed species-level OTU_0.97_ (after subsampling to 44 901 reads per sample; Supplementary Table S2). The contribution of each OTU_0.97_ to individual sand grain communities was variable as estimated by an inverse Simpson index (87±27). Interestingly, the estimated OTU_0.97_ richness was very similar among SSGs (Chao1: 8432±1349) and independent of their grain size. Faith’s phylogenetic diversity ranged between 222 and 326 for the individual sand grains indicating differences in genetic diversity on sand grains.

Beta diversity was studied using UniFrac analysis (Supplementary Table S3). Unweighted Unifrac showed a genetic similarity of 39–50% (mean 45%) between any sand grain community confirming that these are different. Weighted UniFrac analysis considering OTU_0.97_ abundances resulted in a much higher genetic similarity with 50–85% (mean 71%) indicating that less abundant and rare OTU_0.97_ are mainly responsible for the observed genetic differences between sand grains.

The diversity of individual sand grains was compared with the diversity found in bulk sediments. We retrieved 75 134, 129 394 and 137 585 quality-trimmed sequences for samples bulk1 to bulk3 (Supplementary Table S2). Diversity values for the three replicate data sets from bulk sediments were very similar with 6759–6924 observed OTU_0.97_, 13 059–14 155 estimated OTU_0.97_, inverse Simpson indices of 215–230, and Faith’s PD of 348–358. Unweighted and weighted UniFrac similarity values were 54-55% and 93-96%, respectively (Supplementary Table S3).

**Figure 2:**
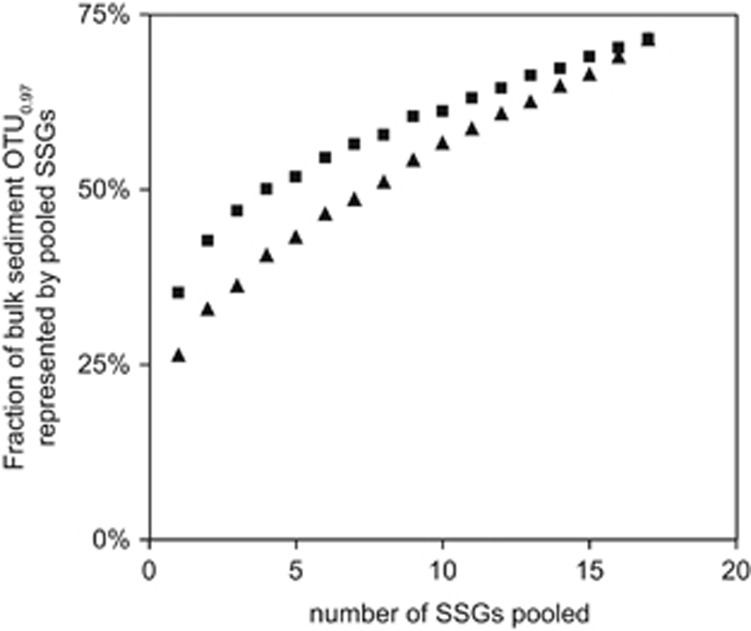
Fraction of bulk sediment OTU_0.97_ richness shared by data sets from pools of SSGs. Depicted values based on consecutively pooled SSGs in decreasing order (▪) or increasing order (▴) of their individual OTU_0.97_ richness. For bacterial diversity indices of individual sand grains see Supplementary Table S2. Subsampling of SSG data sets and bulk sediment data sets were done in a way that always identical number of sequences were compared.

A comparison of bacterial diversity on SSGs and in bulk sediment showed that individual sand grains harbored 27–42% of all OTU_0.97_ present in bulk sediment (Supplementary Table S4). The sand grains’ bacterial communities are as different to each other as to bulk sediments. By sequencing of only four of the most diverse and eight of the least diverse sand grains, already 50% of the OTU_0.97_ richness found in bulk_pooled_ was covered (Figure 2). By sequencing of 17 sand grains, we covered 71% of OTU_0.97_ found in bulk_pooled_, thus describing the major part of total diversity in several grams of sediment. This is in contrast to findings from soils where observed bacterial richness on macroaggregates (size: 250–1000 μm) was 3- to 27-fold lower than in bulk soil (Bailey *et al.*, 2013). Soil is characterized by an uneven nutrient distribution and limited exchange of bacterial community members between macroaggregates. In marine sediments, in contrast, a connected water-saturated pore space enables a constant and even nutrient supply and cell dispersal allowing the establishment of more similar bacterial communities on individual sand grains than on individual soil macroaggregates.

To study the influence of ‘rare biosphere’ organisms on the bacterial diversity on sand grains, we performed the same analysis limited to OTU_0.97_ contributing >0.1 and >1 per mill (Supplementary Table S4). The selected thresholds were based on the definition for ‘rare biosphere’ given by Galand *et al.* (2009) and Pedrós-Alió (2012), respectively. Excluding the rare biosphere, the vast majority of the bacterial community in bulk sediment was represented on each SSG (OTU_0.97_ >0.1‰: 60±6% OTU_0.97_ >1‰: 83±4%).

### Core community on sand grains

**Figure 3:**
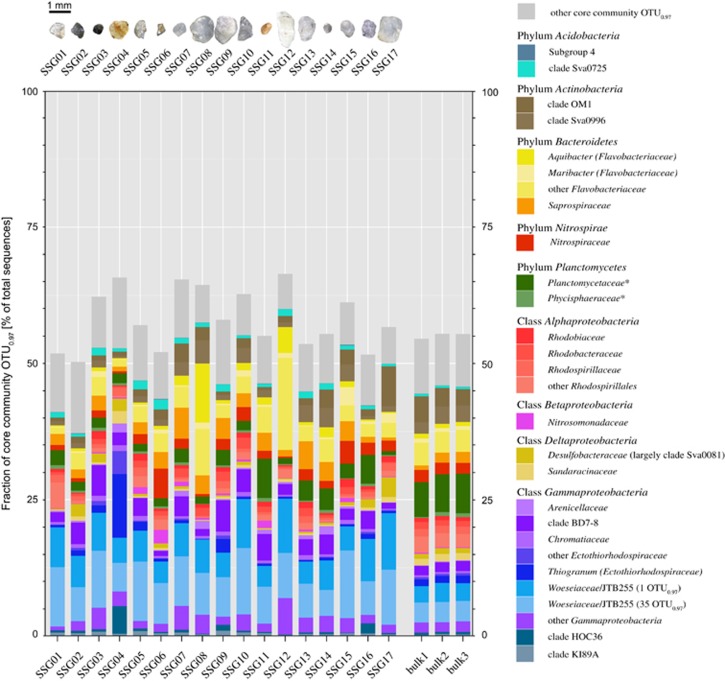
Relative contribution of sand grain core community OTU_0.97_ to total community based on sequencing of 16S rRNA gene fragments. The core community that was present on all 17 individual sand grains in June 2016, comprised 394 OTU_0.97_. The taxonomic classification of core community OTU_0.97_ is given on family level. Thus, each depicted family can comprise several OTU_0.97_. Core community families and uncultivated clades contributing on average <0.5% to total 16S rRNA gene sequences are summarized as 'other core community OTU_0.97_'. Depicted core community composition is based on subsampled data sets (*n*=44 901 sequences). For some families, that is, *Woeseiaceae*/JTB255, *Flavobacteriaceae* and *Ectothiorhodospiraceae*, a higher taxonomic resolution is given. *Sequences classified as *Planctomycetaceae* and *Phycisphaeraceae* rather represent several unclassified families within the class *Planctomycetia* and *Phycisphaerae*, respectively.

We identified a core community that was present on all 17 sand grains comprising 394 OTU_0.97_. Although these core sequences represented only 1.7% of the total observed OTU_0.97_ on sand grains, they made up one-half to two-thirds of total sequences retrieved from each sand grain (Figure 3). A large core community is a common phenomenon observed for marine microbial communities. Seasonal sampling at the long-term monitoring site L4 in the English Channel (Gilbert *et al.*, 2009) or at a tidal sandy beach in the North Sea (Gobet *et al.*, 2012) both showed a resident core community comprising only 0.5% of the total OTU_unique_ diversity but 50% of the sequence reads or represented the most abundant OTU_unique_.

Taxonomic classification of the 394 core community OTU_0.97_ revealed 82 family-level clades (Figure 3). Major core community members were gammaproteobacterial *Woeseiaceae*/JTB255 (10–21% of total sequences retrieved from a SSG), *Ectothiorhodospiraceae* (0.1–16%), and clade BD7-8 (0.9–6%), bacteroidetal *Flavobacteriaceae* (2–23%) and *Saprospiraceae* (0.6–6%), planctomycetal *Planctomycetia* (0.7–7%) and *Phycisphaerae* (0.1–0.7%), deltaproteobacterial *Desulfobacteraceae* (0.1–4%) and *Sandaracinaceae* (0.1–2%), acidobacterial clade Sva0725 (0.5–2%), actinobacterial clades OM1 (0.6–7%) and Sva0996 (0.4–4%), alphaproteobacterial *Rhodobiaceae*, *Rhodobacteraceae* and *Rhodospirillaceae* (each 0.2–2%), as well as *Nitrospiraceae* (0.3–5%) of the phylum *Nitrospira*. The large core community supports our findings that most differences between bacterial communities on individual sand grains can be explained by the ‘rare biosphere’. However, when looking at higher taxa and considering the abundant community members, sand grains are similar. Their genetic diversity and therefore metabolic diversity approaches that of bulk sediments. Based on the phylogenetic affiliation, we have first indications that all major elemental cycles are present, however, this needs to be backed up by metagenomic sequencing.

### Bacterial non-core community on sand grains

On each sand grain, the non-core community comprised few thousands of OTU_0.97_ contributing one-third to one-half of total sequences (Supplementary Figure S5) suggesting an enlarged genetic potential of the bacterial community on sand grains. Although the non-core community did not comprise additional major phyla, 290 more family-level clades were detected. In bulk sediments, additional 257 family-level clades were detected that were not represented by the sand grain core community.

Two major quantitative differences between detected SSG and bulk sediment communities were observed: *Woeseiaceae*/JTB255 were more frequently retrieved from SSG compared with bulk sediments (average 15 vs 6% of total sequences). Vice versa, *Planctomycetes* were more abundant in the data sets from bulk sediments (2 vs 7%). These mismatches may be explained by different efficiencies of cell lysis during SSG-PCR and the extraction procedure of DNA from bulk sediments.

### *In situ* identification of microbial communities on sand grains

CARD-FISH on sand grains enabled the identification of phylogenetically diverse clades directly in their natural habitat. The greatest advantage is that cells do not need to be dislodged from grains, which is the standard procedure for hybridization of microbial populations in sandy sediments (Ishii *et al.*, 2004). Therefore, CARD-FISH on sand grains allows the detection of microbe–microbe associations not only in tight aggregates that may sustain sonication.

**Figure 4:**
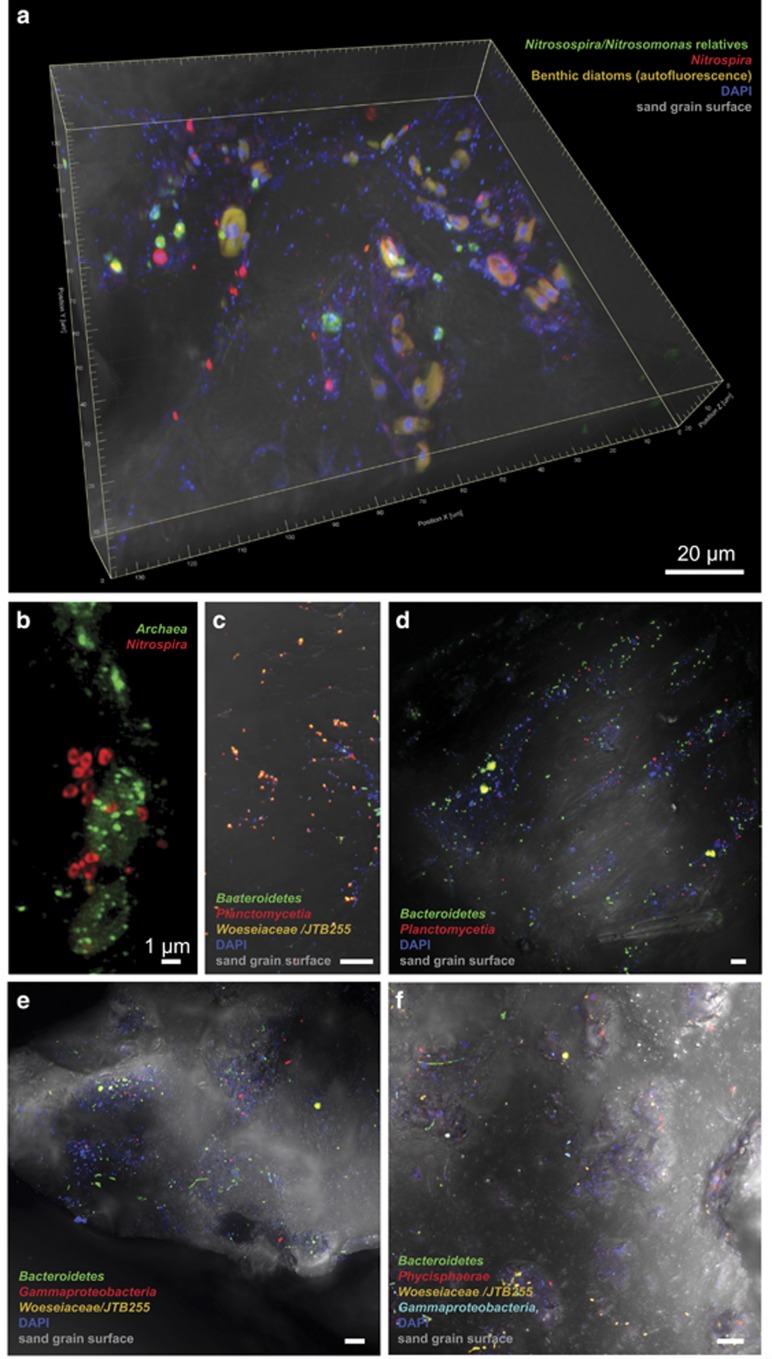
Direct visualization of taxa on sand grains using CARD-FISH and confocal laser scanning microscopy. Targeted taxa are indicated in the individual panels **a** to **f**. DAPI signal (in blue) shows all cells not targeted by the probes. All images (except for **b**) are composite micrographs of fluorescent signals and transmitted light of the sand grain’s surface. Micrograph a is also available as a video in the Supplementary Information showing the microbial colonization of protected and exposed areas in the three-dimensional space. Micrograph **b** is a superresolution structured illumination image (SR-SIM). If not otherwise indicated, scale bar refers to 10 μm. Probes used are listed in Supplementary Table S1.

Numerous large cells were observed on the grains that colonized nearly exclusively the protected areas with a macrotopography. These cells showed autofluorescence at >500 nm upon excitation at 488 or 561 nm (Figure 4a, video available in Supplementary Information). Using the *Eukarya-*specific probe EUK561, these cells were identified as eukaryotic. Based on morphology and 16S rRNA gene sequences that were classified as chloroplasts, these microalgae are mostly diatoms showing a high species diversity. In very close proximity, we found diverse *Bacteria* and *Archaea*.

The detection of any possible colocalization is a particular strength of CARD-FISH on sand grains. Here, we focused on the detection of colocalizing ammonia- and nitrite-oxidizing *Bacteria* and *Archaea* known to form separate, dense clusters, which were in contact with each other, for example, in waste water treatment plants (Schramm *et al.*, 1998; Daims *et al.*, 2001). Based on the 16S rRNA gene sequences from the sand grains and from metatranscriptomic data from the same study site (Probandt, unpublished data), candidates for ammonia oxidation were archaeal *Thaumarchaeota* (mainly '*Candidatus* Nitrosopumilus'), as well as *Betaproteobacteria* of the *Nitrosomonadaceae* (Figure 3). Candidates for nitrite oxidation were *Nitrospiraceae,* mainly *Nitrospira*-related organisms (Watson *et al.*, 1986). Some *Nitrospira* have recently been reported to completely oxidize ammonia (comammox) to nitrate independent of any microbial partners (Daims *et al.*, 2015; van Kessel *et al.*, 2015). Phylogenetic analyses of sand grain *Nitrospira* sequences, however, showed only 83–89% similarity and a distant clustering to comammox bacteria. Using existing and newly developed probes (Supplementary Table S1) small, separate aggregates of *Nitrosospira* and *Nitrospira* (Figure 4a) or *Archaea* and *Nitrospira* (Figure 4b) were detected that, in general, consisted of about 3–10 cells. Also single cells of each group were detected on the sand grains. The archaeal aggregates were further identified as *Thaumarchaeota* using probe Cren537. Compared with activated sludge and biofilms in waste water treatment plants where both partners occur as densely packed, larger aggregates, partners on sand grains were detected in smaller aggregates. These were sometimes in close contact with their partner aggregates, but often more distant to each other with cell–cell distances of 0 and 11 μm between close and distant *Archaea* and *Nitrospira* and 0 and 51 μm between close and distant *Nitrosospira* and *Nitrospira*. This may be explained by the characteristics of permeable sediments, in which intermediates are transported advectively through the sediment matrix. Some *Nitrospira* are also capable of mixotrophy being heterotrophs some of the time, which could also explain the lack of co-localization with ammonia-oxidizers (Koch *et al.*, 2015).

By far the majority of cells on sand grains could be identified as *Bacteria*. Based on the sand grain core community, we used a set of specific oligonucleotide probes targeting the most abundant benthic bacterial clades such as *Planctomycetia, Phycisphaerae, Gammaproteobacteria, Bacteroidetes* and *Woeseiaceae*/JTB255. Colonization pattern of targeted core community taxa was rather scattered on sediment grains. *Gammaproteobacteria* (including *Woeseiaceae*/JTB255), *Planctomycetes* and *Bacteroidetes* (Figures 4c–f) were most abundant. Members of these clades were observed as single cells or microcolonies. Rod-shaped or coccoid *Woeseiaceae*/JTB255 cells populated most sand grains accounting for estimated 5% of total cells. This supports previous quantifications done on membrane filters showing an abundance of 3–6% of total cells in several coastal surface sediments (Dyksma *et al.*, 2016). Cells identified as *Planctomycetia* were found in close association with benthic microalgae. Here, heterotrophic *Planctomycetia* may profit from polysaccharides (Hoagland *et al.*, 1993). However, numerous *Planctomycetia* were also found isolated from indents where organic substrates usually accumulate.

In conclusion, each sand grain investigated in this study was the habitat for around 10^5^ cells representing several thousand species. The average distance between any two cells on a sand grain in protected areas was 0.5±0.7 μm and therefore 100-fold shorter than the average distance between cells in the water column. Confirming our original hypothesis, the bacterial diversity on species level differed between SSGs as shown by presence/absence and genetic distances of species. However, when looking at higher taxa and considering the abundant community shared by any sand grain, sand grains are quite similar and provide the habitat for numerous higher taxa indicative for functional groups.

In contrast to stable soils, the dynamic and permeable surface sediments are characterized by fluctuating redox conditions and substrate availabilities resulting in diverse and versatile microbial communities. Our data suggest that each sand grain is a small microbial repository from which the major cycles of carbon, nitrogen and sulfur transformations typical of marine benthic habitats could be reconstituted.
