# Supplementary material for: Microbial life on a sand grain: from bulk sediment to single grains
Source: ISME J. 2017 Dec 1;12(2):623–33. doi: 10.1038/ismej.2017.197 (PMC5776476; doi:10.1038/ismej.2017.197)
Supplement: Supplementary Material [file ismej2017197x1.pdf]

Microbial life on a sand grain: from bulk sediment to single grains

Probandt, D[1]., Eickhorst, T.[1,2], Ellrott, A. [1], Amann, R.[1], and Knittel, K.[1*],

[1]Max Planck Institute for Marine Microbiology, 28359 Bremen, Germany

[2]University of Bremen, Faculty 2 (Biology/Chemistry), 28359 Bremen, Germany

Supplementary Information

*corresponding author

Katrin Knittel, Max Planck Institute for Marine Microbiology, Dept. Molecular Ecology, Celsiusstraße 1, 28359 Bremen, Germany

email: kknittel@mpi-bremen.de

Phone: +49 421 2028935

Supplementary Methods

Micro-computed tomography (µCT)

For micro-computed tomography, we subsampled the centre of undisturbed sediment from large push cores (diameter 40 mm) with a polyethylene (PE) cylinder (14 mm diameter × 30 mm height). Cylinders were gently pushed through an alignment adapter allowing a centered and straight generation of subsamples. The filled PE cylinders were placed on a permeable bandage and fixed on a custom-made rack in a sealed plastic container to prevent tipping over and allow liquid exchange from the bottom of the samples. The cylinders were filled with glass beads from the top to minimize resuspension effects of the sediment surface. Samples in PE cylinders were dehydrated in a graded series of acetone (70%, 80%, 90%, 2 × 100% acetone). All solutions were added to the bottom of the container ensuring the entire replacement of the pore space. Samples in PE cylinders were resin impregnated in a desiccator under vacuum (220 mbar) using a polyester resin as previously described (Eickhorst and Tippkötter, 2008). After polymerization (28 days) samples were removed from the PE cylinder and the base was polished orthogonal to the sampling axis. X-ray µ-CT visualization was performed using scanning facilities at the Department of Geosciences, University of Bremen, Germany (CT-ALPHA, ProCon, Germany). Scan settings were optimized for the visualization of the resin-filled pore space and the inorganic sediment matrix at a resolution of 6.216 µm per voxel. Radiographs were reconstructed into a three-dimensional volume using VOLEX (Fraunhofer IIS, Germany) and volume rendering and image extraction was done using Avizo 9.0.1 (FEI, USA). Horizontally orientated µCT 2D images were subjected to local means 2D filter (Sigma 5) using FIJI. Stack dataset was analyzed using AMIRA (v. 6.3, FEI, The Netherlands) after manual

thresholding and segmentation into clearly separable pore space and solid phase. Solid phase surface was calculated and related to one cm^3. For analysis of sediment porosity, grey-scale image stacks were segmented into binary images and processed with the fully automated adaptive window indicated Kriging algorithm as described (Houston et al., 2013).

Calculations of cell density, colonized surface area and cells per sand grain

The footprint of an average microbial cell was 0.43 μm^2. The calculation is based on an estimated community composition of 50% small and 5% large cocci (diameter, on average 0.5 µm and 1 µm, respectively) and 30% small and 15% large rod-like cells (0.5 µm × 1 µm and 0.5 µm × 2 µm, respectively). The footprint is used together with the colonization density (cells cm^{-2}) to determine the colonized fraction of the grain surface. Cell numbers per sand grains were calculated according to equation I:

Eq. I $$\text{cells per sand grain} = \text{grain surface area } [cm^2] \times \text{colonization density } [cm^{-2}]\ ,$$

where grain surface area is 1.3×10^{-3} cm^2 and 13×10^{-3} cm^2, respectively, when assuming a perfect sphere with a diameter of 202 µm and 635 µm (size range for 80% of sand grains, n= 199)

or to equation II and III

Eq. II $$\text{cells per sand grain} = \frac{\text{cells per cm}^3\text{ sediment}}{\text{no.grains per cm}^3\text{ sediment}}$$

Eq. III $$\text{no. grains per cm}^3 = \frac{\text{volume of sediment } [cm^3] \times (1 - \text{porosity})}{\text{volume of sand grain } [cm^3]}\ ,$$

where porosity is 0.4 and grain volume is 0.43×10^{-5} cm^3 (202 µm diameter) and 13×10^{-5} cm^3 (635 µm diameter).

Cell-cell distance measurements

Image z-stacks of three different sand grains stained with SYBR green I were obtained via confocal laser scanning microscopy (LSM 780, Carl Zeiss Microscopy GmbH, Germany) and visualized using a 3D maximum intensity projection (Imaris x64, Bitplane AG, Switzerland). We defined two types of areas on the sand grains based on their surface topology: i) exposed areas characterized by mainly convex and smooth surfaces with some microtopography and ii) protected areas with micro- and macrotopography (Supplementary Figure S4).

In total, seven regions of interest (ROI) of exposed areas and eleven ROI of protected areas from three different sand grains were analyzed with the software ACMEtool 3 (July 2014; M. Zeder, Technobiology GmbH, Switzerland). The coordinates and area of each valid object was used to calculate a distance matrix for all identified valid objects per ROI (Supplementary Figure S3). Assuming all cells being spheres, the cell radius was estimated from the object area according to equation IV

Eq. IV $$radius = \sqrt{\frac{area}{\pi}}$$

The object's central point was calculated from the bounding boxes' vertices' x- and y-coordinates according to equation Va and Vb.

Eq. Va $$center_x = left + \frac{right - left}{2}$$

and

Eq. Vb $$center_y = top + \frac{bottom - top}{2}$$

And the cell-cell distance according to **Eq. VI**

$$distance_{1,2} = \sqrt{|center_{x2} - center_{x1}|^2 + |center_{y2} - center_{y1}|^2} - radius_1 - radius_2$$

Only the closest neighbor was considered for calculations. Furthermore, only non-touching cells were included into the analysis thus the obtained values are overestimating the actual mean cell-cell distances. Applying this model on cell morphologies different from cocci/spheres, cell-cell distances would be under- or overestimated depending on their x/y/z-orientation to any other cell.

CARD-FISH on sand grains

Although SYBR green I staining of total cells showed bright signals, it was necessary for CARD-FISH to replace SYBR green I by DAPI. The green-emitting dyes were needed for tyramide labeling due to their high sensitivity. Up to four different HRP-labeled probes could successfully be applied in consecutive hybridizations without noticeable loss of signal intensity. Monolabeled and tetralabeled probes (except for *Planctomycetia*) did not work on sand grains due to too low brightness. Used tyramides were labeled with the standard dyes Alexa488, Alexa594 or Alexa647. For visualization of a fourth target group, we mixed Alexa488- and Alexa594-tyramides in an equimolar ratio for amplification, resulting in mixed-color signals. All targeted cells showed a comparably bright signal with both dyes, therefore, false positive cells showing only either of the colors can be excluded.

Quality trimming and sequence processing

Paired-end reads were quality trimmed (>q21, both ends) and merged (strict, overlap 20) using software package BBmap (v. 36.92) at high confidence settings. A subset of 100.000 sequences per sample was further processed according to the MiSeq SOP (Kozich et al., 2013) with mothur v.1.39.5 (Schloss et al., 2009; Westcott and Schloss, 2017). For removal of potential artificial diversity, a pre-clustering at 99% sequence

identity and *de novo*-based chimera removal using UCHIME (Edgar et al., 2011) was performed. Afterwards, sequences were classified using the SILVA database SSU Ref NR, release 123 (Quast et al., 2013). All sequences that were classified as *Archaea*, chloroplast 16S rRNA or non-16S rRNA were removed from the dataset because they resulted from an unspecific PCR amplification and therefore do not represent the real diversity of *Archaea* and *Eukarya* on a sand grain or in bulk sediments. Remaining sequences were globally clustered in operational taxonomic units (OTU) at 97% similarity using the OptiClust algorithm. Finally, rare OTU$_{0.97}$ that were represented by ≤2 sequences in the whole dataset, i.e. single sequence OTU absolute (SSO$_{abs}$), and double sequence OTU absolute (DSO$_{abs}$), were removed prior to diversity analysis. The removal of SSO$_{abs}$ and DSO$_{abs}$ has been done to reduce artificial diversity/noise as previously suggested for Illumina 16S rRNA gene fragment data (e.g. Allen et al., 2016). Removed SSO$_{abs}$ and DSO$_{abs}$ contributed each at maximum only 1 sequence of 100.000 sequences in the entire data set. To test whether the removal of SSO$_{abs}$ and DSO$_{abs}$ from the whole dataset affected further statistical tests, we performed Procrustes correlation analysis (Gower, 1975) based on three-dimensional non-metric multidimensional scaling (NMDS) of the Bray-Curtis dissimilarity coefficient (Bray and Curtis, 1957) of two data sets. One data set containing all OTU$_{0.97}$ and one data set without SSO$_{abs}$ and DSO$_{abs}$. The correlation of the two ordinations was highly significant (Procrustes correlation coefficient = 0.95, p=0.001, 999 permutations), indicating that the removal of SSO$_{abs}$ and DSO$_{abs}$ did not affect the overall trend. In the subsampled data set (44,901 sequences per sample) used for alpha diversity analysis, relative single sequence OTU and double sequence OTU (SSO$_{rel}$ and DSO$_{rel}$) still remained in the database and thus are considered in the Chao1 estimator of total richness (Chao 1984).

Diversity analysis

For analysis of the alpha and beta diversity, the data sets were subsampled to lowest number of sequences in the total data set (N=44,901).

Alpha diversity analysis was performed based on phylotype- (OTU) and phylogenetic-based methods. Inverse Simpson (Simpson, 1949) and Chao1 (Chao, 1984) were independently calculated 25 times using the R-package vegan (v.2.4, Oksanen et al., 2013) and customized R-scripts with $OTU_{0.97}$ and are displayed as a mean value. For calculation of Faith's phylogenetic diversity (Faith, 1992), a phylogenetic tree of $OTU_{0.97}$ representative sequences of the subsampled data set (N=44,901) was reconstructed using FastTree2 (Price et al., 2010) implemented in the ARB software package (Ludwig et al., 2004). Based on the phylogenetic tree, Faith's PD was calculcated using the R-packages ape (v.4.1, Paradis et al., 2004) and picante (v. 1.6, Kembel et al., 2010).

Beta diversity analysis was performed based on phylotypes ($OTU_{0.97}$) and their phylogenetic affiliation. To assess the (phylo)genetic distance between single sand grain's and replicate bulk sediment samples' bacterial communities, unweighted and weighted UniFrac distance matrices (Lozupone and Knight, 2005) were calculated based on the reconstructed phylogenetic tree using the R-package GUniFrac (v.1.0, Chen, 2012).

For quantification of the nestedness of the bacterial diversity from individual sand grains in the bulk sediments, occurrences of observed bulk sediment $OTU_{0.97}$ on sand grains were calculated. Therefor bulk_1 to bulk_3 data were pooled and compared to each single sand grain.

Supplementary Results

Relative abundance and phylogeny of nitrifying *Bacteria* & *Archaea* and chloroplast 16S rRNA gene tag sequences

10.000 quality-trimmed and merged sequences from each sample (17 sand grains, 3 bulk sediments) were submitted to the SILVAngs pipeline (Quast et al., 2013; database release 128). As output, SILVAngs provides aligned sequences which can be readily imported into the arb software package (Ludwig et al., 2004; Pruesse et al., 2007). In total, 2 ±2% retrieved from the 17 sand grains and 2 ±0.7% of total sequences retrieved from bulk sediments were assigned to ammonia- and nitrite-oxidizing bacteria. Most abundant were sequences affiliated with betaproteobacterial *Nitrosomonadales* (0.6 ±0.7% of total bacterial sequences retrieved from single sand grains and 0.2 ±0.1% from bulk sediments) and *Nitrospirae* (2 ±1% and 2 ±0.1%). Only of minor relative abundance were *Nitrospina* spp. (0.1 ±0.1% and 0.1 ±0.02%). Gammaproteobacterial *Nitrosococcus* and alphaproteobacterial *Nitrobacter* were found only sporadically on one sand grain in sequence abundances <0.01%. The fraction of archaeal sequences were 0.4 ±0.4% and 0.3 ±0.1% of total sequences retrieved from single sand grains and bulk sediments, respectively. Archaeal sequences were not further analyzed because their amplification during PCR resulted from unspecific primer binding thus not covering the archaeal diversity. More than 99.9% of retrieved archaeal sequences were affiliated with *Thaumarchaeota* and *Woesearchaeota* DHVEG-6.

For phylogenetic analysis, sequences were added to the tree provided in SILVA database release 128 under parsimony criteria without allowing changes in the overall tree topology. In total, sequences affiliated with the order *Nitrosomonadales* formed 314 $OTU_{0.97}$. The tree was optimized using Parsimony interactive using the heuristic

optimizer (global optimization). All sequences were only distantly related to ammonia-oxiding *Nitrosomonas* with 82.0-92% sequence similarity but closely related to *Nitrosopira multiformis / Nitrosovibrio tenuis* with 92.0-98.3% sequence similarity suggesting a common genus if not species (Yarza et al., 2014).

Sequences affiliated with *Nitrospirae* clustered into 1064 $OTU_{0.97}$. More than 99% affiliated with *Nitrospira marina* (93-96% sequence similarity) suggesting that they derived from organisms of the same genus. They were only distantly related to "*Candidatus* Nitrospira nitrificans" and "*Candidatus* Nitrospira nitrosa" (83-89%) that have been described to be able of complete nitrification of ammonia to nitrate (comammox; Daims et al., 2015; van Kessel et al., 2015).

Plastid 16S rRNA gene sequences from chloroplasts made up 3 ±3% of total sequences retrieved from the sand grains and 9 ±2% retrieved from bulk sediments. All of them affiliated with sequences from *Bacillariophyceae* indicating that marine diatoms were the dominant microalgae on sand grains. A more specific taxonomic assignment was not possible based on their plastid 16S rRNA gene sequences.

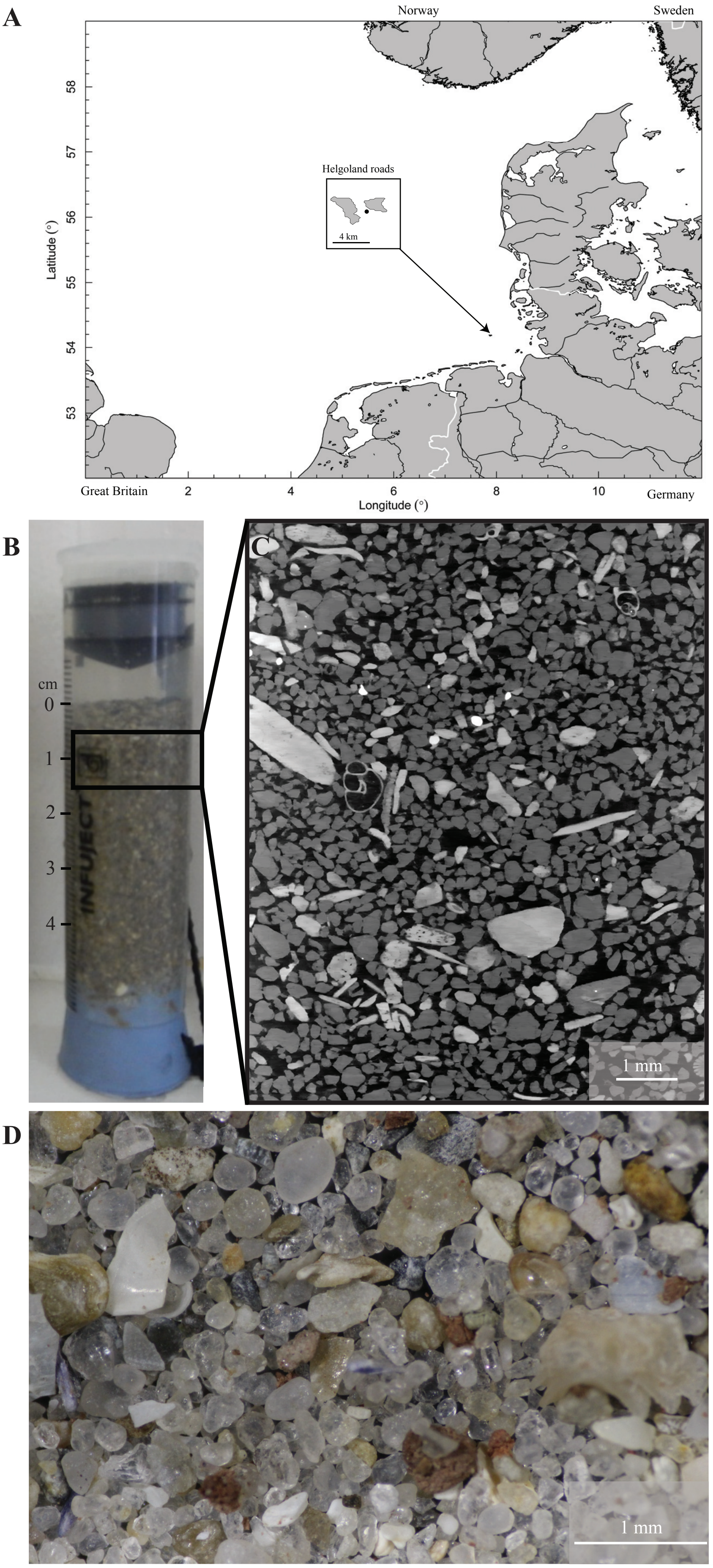

Figure S1. Sublittoral surface sediment at site Helgoland Roads.
A, Geographic location of sampling site Helgoland Roads in the southern North Sea. The close up shows the location of Helgoland Roads between the islands of Helgoland and Helgoland-Düne. B, sediment push core. C, reconstruction of sediment vertical section using µCT images. Pore space (black), solid phase composed of sand grains (dark to light grey) or shell debris (light grey, sharp edges). Shown section corresponds to a sediment depth of 0.5 cm to 1.5 cm below seafloor. D, binocular photograph of dried sand.

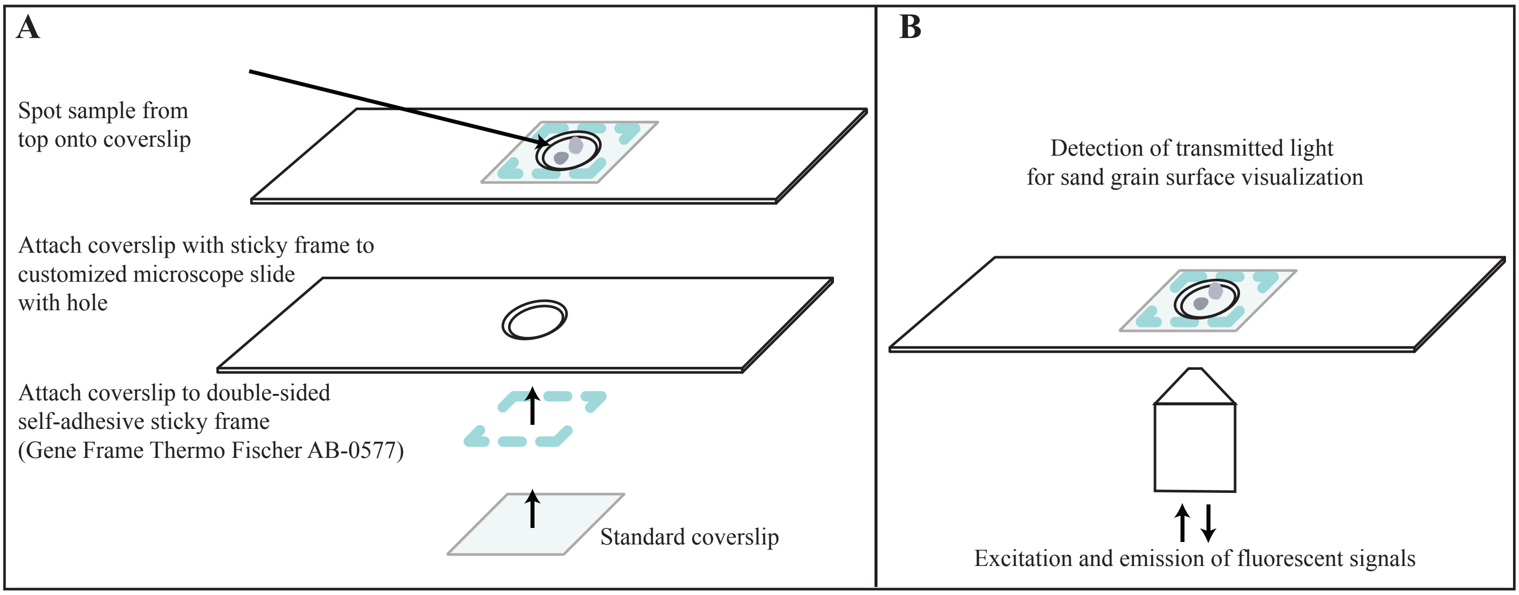

Figure S2: Schematic drawing of customized glass slide for visualization of microbial cells on sand grains using inverse confocal laser scanning microscopy.

A, slide preparation. A hole was drilled carefully using a standard household diamond drill. The sand grain was spotted through the hole on the cover slip and allowed to dry. Since the experimental set-up is not a closed system (air-contact to the top), heat-induced motion during long image acquisition was minimized. B, sample visualization using the inverse microscope.

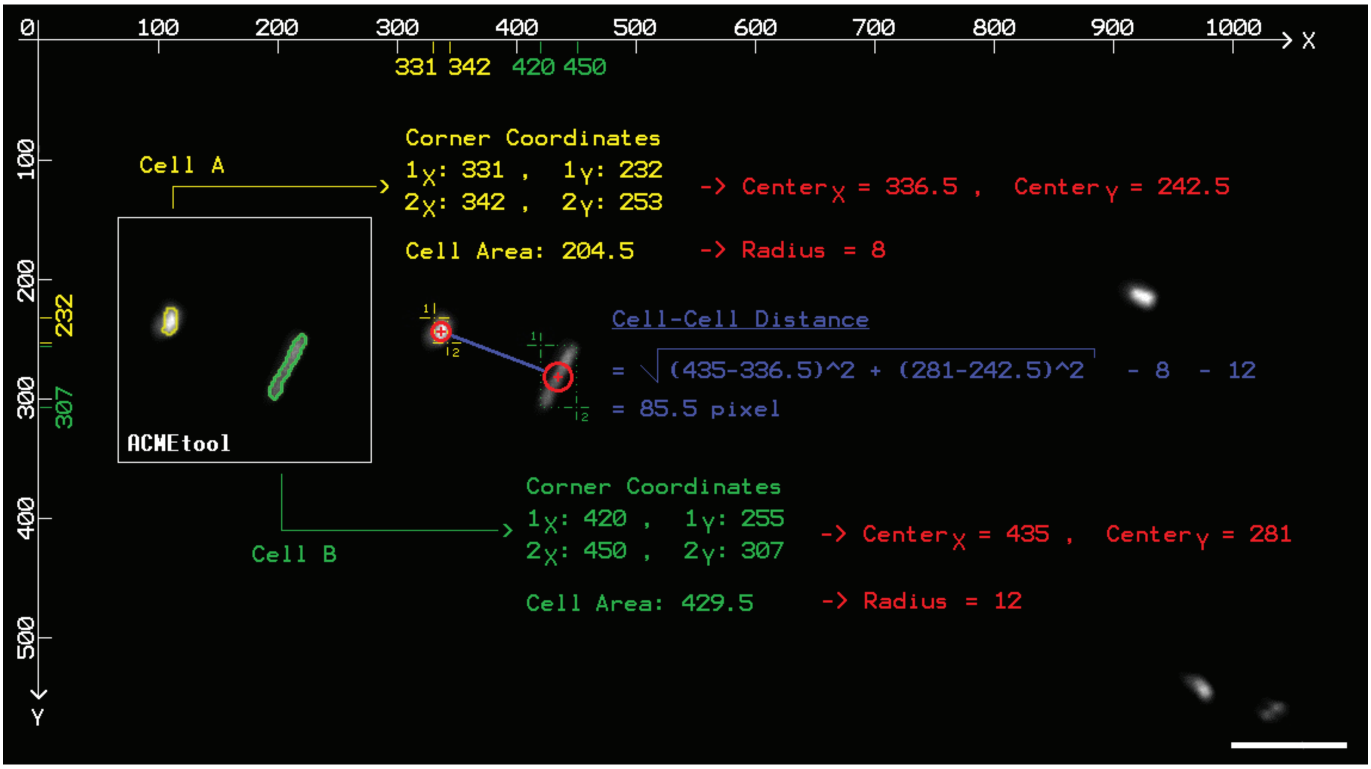

Figure S3. Schematic diagram on cell-cell distance calculation.
Step 1: Cell detection using ACMEtool (white box). Step 2: Export of relevant cell features for further analysis (cell A shown in yellow, cell B shown in green). Step 3: Calculation of center point and radius for each cell (results shown in red color). Step 4: Cell-cell distance calculation (blue color). The calculation uses a pixel-based coordinate system. The scale bar corresponds to 7 µm.

Figure S4

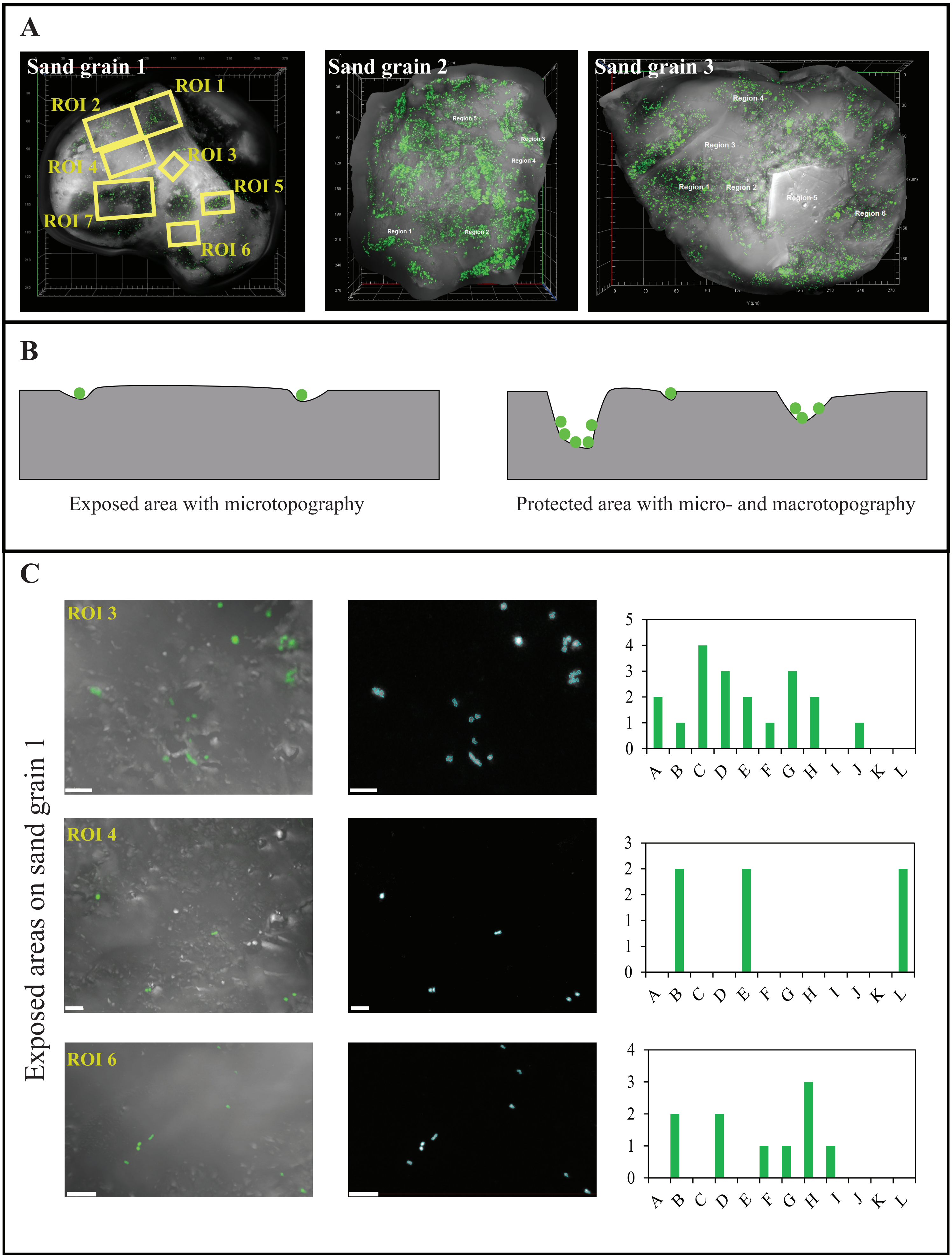
A
Sand grain 1
ROI 1
ROI 2
ROI 3
ROI 4
ROI 5
ROI 6
ROI 7
Sand grain 2
Region 1
Region 2
Region 3
Region 4
Region 5
Sand grain 3
Region 1
Region 2
Region 3
Region 4
Region 5
Region 6
B
Exposed area with microtopography
Protected area with micro- and macrotopography
C
Exposed areas on sand grain 1
ROI 3
ROI 4
ROI 6
A B C D E F G H I J K L

Figure S4, continued

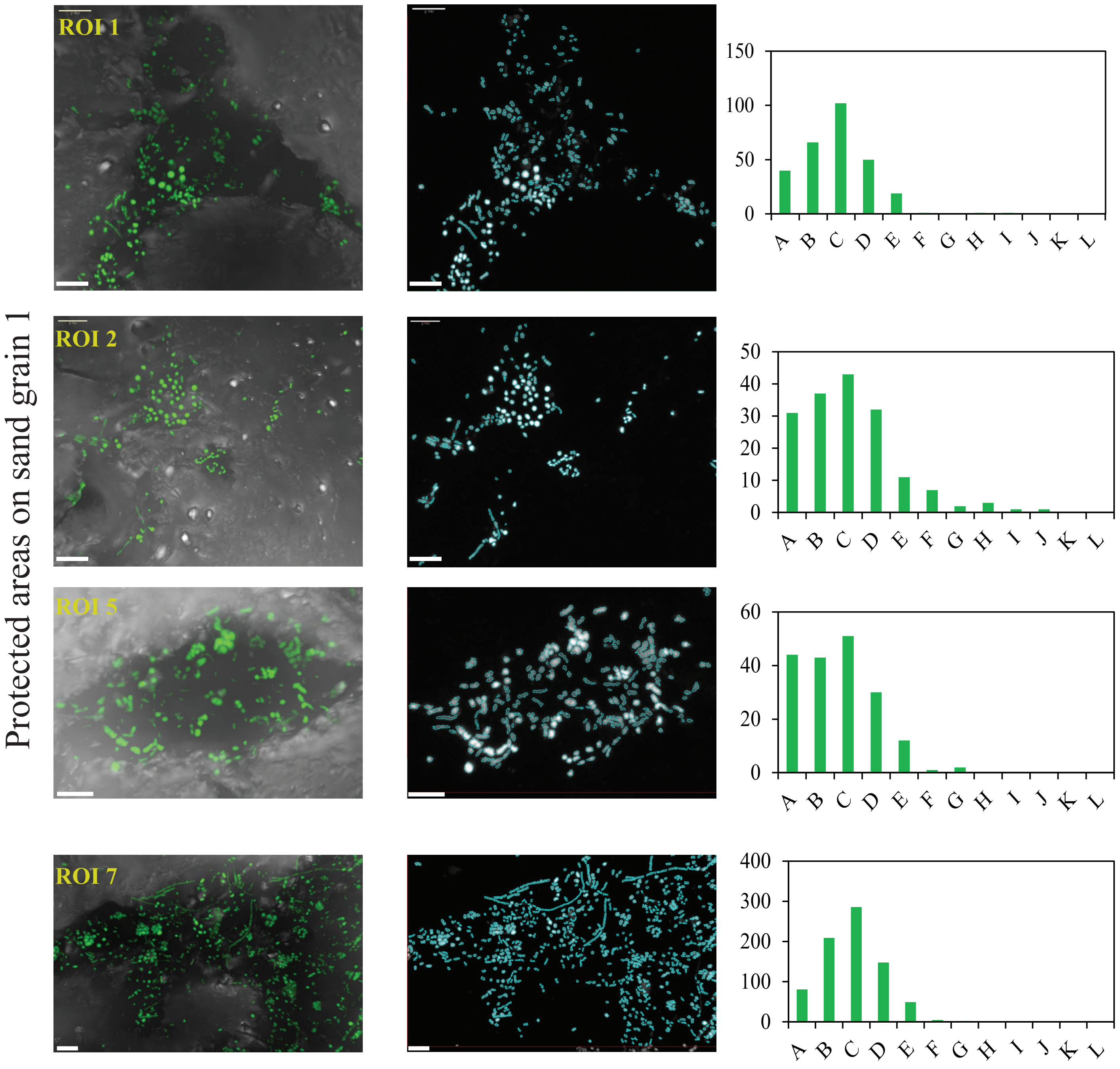

Cell-cell distance categories

	A	B	C	D	E	F	G	H	I	J	K	L
µm	≤ 0.125	> 0.125-0.25	> 0.25-0.5	> 0.5-1	> 1-2	> 2-3	> 3-5	> 5-7	> 7-10	> 10-15	> 15-20	> 20-30

Figure S4: Cell-cell distances in exposed and protected areas on sand grains.
A, three representative sand grains stained with SYBR green I were analyzed for cell-cell distances in selected regions. B, schematic cross section of (left) an exposed area characterized by mainly convex and smooth surfaces with some microtopography and (right) protected areas with micro- and macrotopography. Green dots symbolize cells colonizing the sand grain's surface. C, exemplary for grain 1, regions of interest (ROI) of exposed (ROI 3, 4, 6) and protected (ROI 1, 2, 5, 7) areas and corresponding cell-cell distance measurements are shown. Left, micrograph of original ROI; middle, cell detection by ACMEtool; right, minimum cell-cell distance distribution as shown in distance classes. See key for distances in µm. Scale bar, 5 µm.

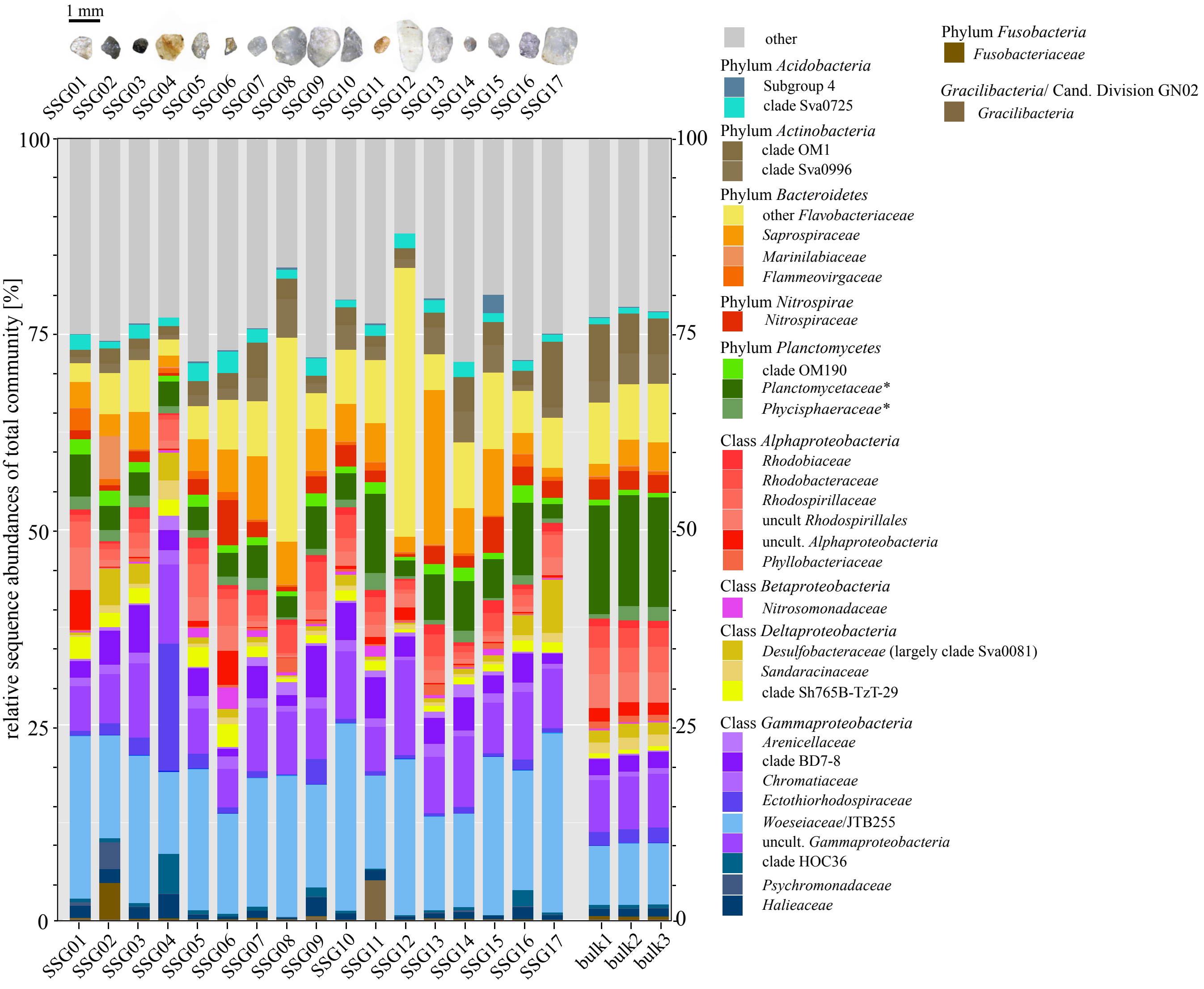

Figure S5: Bacterial community composition on single sand grains and in bulk sediment as shown by Illumina partial 16S rRNA gene sequencing.
Depicted are the 10 most sequence-abundant family-level clades found on the individual sand grains or in bulk sediment datasets. Remaining family level clades are summarized as "other". Clades described as "uncultured (uncult)" could not be further classified and might contain numerous $OTU_{0.97}$ of respective taxon level. Depicted community composition is based on subsampled datasets (N=44,901). * Sequences classified as *Planctomycetaceae* and *Phycisphaeraceae* rather comprise several unclassified families within the class *Planctomycetia* and *Phycisphaerae*, respectively.

Table S1. Oligonucleotide probes used for CARD-FISH and FISH.

Probe name	Target	Sequence (5' - 3')	FA [%][1]	Target	Reference
EUB338 I		GCT GCC TCC CGT AGG AGT	35	16S rRNA	Amann et al., 1990
EUB338 II	Most *Bacteria*	GCA GCC ACC CGT AGG TGT	35	16S rRNA	Daims et al., 1999
EUB338 II		GCT GCC ACC CGT AGG TGT	35	16S rRNA	Daims et al., 1999
ARCH915a	*Archaea*	GTG CTC CCC CGC CAA TTC CT	35	16S rRNA	Stahl and Amann, 1991
CREN537	Marine Group I *Thaumarcheota*	TGA CCA CTT GAG GTG CTG	20	16S rRNA	Teira et al, 2004
EUK516	*Eukarya*	ACC AGA CTT GCC CTCC	0	18S rRNA	Amann et al., 1990
NON338	nonsense probe	ACT CCT ACG GGA GGC AGC	35	16S rRNA	Wallner et al., 1993
GAM42a[2]	*Gammaproteobacteria*	GCC TTC CCA CAT CGT TT	35	23S rRNA	Manz et al., 1992
GAM42a_T1038_G1031[2]	*Xanthomonadaceae*	GCC TTT CCA CAT GGT TT	35	23S rRNA	Siyambalapitiya and Blackall, 2005
GAM42a_T1038[2]	*Xanthomonadaceae*	GCC TTT CCA CAT CGT TT	35	23S rRNA	Siyambalapitiya and Blackall, 2005
BET42a[2]	*Betaproteobacteria*	GCC TTC CCA CTT CGT TT	35	23S rRNA	Manz et al., 1992
JTB1270	*Woeseiaceae*/JTB255	GAG CTT TAA GGG ATT AGC GCA CCA	40	16S rRNA	Dyksma et al., 2016a
hJTB1270	Unlabeled helper oligo, used with JTB1270	TTG CTG GTT GGC AAC CCT CTG TAT	40	16S rRNA	Dyksma et al., 2016a
CF968	*Bacteroidetes*	GGT AAG GTT CCT CGC GTA	30	16S rRNA	Acinas et al., 2015
NTSPA712	*Nitrospirae*	CGC CTT CGC CAC CGG CCT TCC	50	16S rRNA	Daims et al., 2001
cNTSPA712	Unlabeled competitor used with NTSPA712	CGC CTT CGC CAC CGG TGT TCC		16S rRNA	Daims et al., 2001
NM645[3]	*Nitrosospira, Nitrosovibrio,* some *Nitrosomonas*, uncultured *Nitrosomonadaceae*	GCC ACA CTC TAG YCT TGT	20-30	16S rRNA	This study
c1NM645	Unlabeled competitor used with NM645	GCC ACA CTC TAG CCT TGC		16S rRNA	This study
c2NM645	Unlabeled competitor used with NM645	GCC ACA CTC CAG CCT TGC		16S rRNA	This study
NM478[3]	*Nitrosospira, Nitrosovibrio,* uncultured *Nitrosomonadaceae*, some *Acidobacteria*	TCT TCC GGT ACC GTC AGT A	20-30	16S rRNA	This study
cNM478	Unlabeled competitor used with NM478	TCT TCC GGT ACC GTC AGM A		16S rRNA	This study
PLA46[4]	*Planctomycetes* except *Phycisphaerae*	GAC TTG CAT GCC TAA TCC	30	16S rRNA	Neef et al., 1998
PHYC309	*Phycisphaerae*	AGT GTC TCA GTC CCG ATG CGG CG	35	16S rRNA	Probandt et al., 2017

[1]: formamide concentration in the hybridization buffer.
[2]: Probes GAM42a, GAM42a_T1038_G1031 and GAM42a_T1038 were used as "GAM42a-mix" at a molar ratio of 1:1:1 together with Bet42a as competitor.
[3]: Probe NM645 is recommended to use. It gives brighter signals than NM478 and has no not-target hits.
[4]: Probe Pla46 was used HRP-labeled or directly labeled with four Alexa594 dye molecules using CLICK chemistry

Table S2: Alpha diversity parameters for single sand grains and bulk sediments based on 16S rRNA gene Illumina tag sequencing. Depicted diversity values show the mean of 25 independent calculations. $OTU_{0.97}$ only represented by one or two sequences in the total dataset (SSO_{abs} and DSO_{abs}; ~0.000001% relative sequence abundance) were excluded from analysis. SSO_{abs}, absolute single sequence $OTU_{0.97}$; DSO_{abs}, absolute double sequence $OTU_{0.97}$ Relative single sequence $OTU_{0.97}$ (SSO_{rel}) are $OTU_{0.97}$ that occur only once in the respective sample but are more sequence-abundant in other samples of the entire data set.

sample	Quality reads [No.]	Subsampled to 44,901 reads each sample: observed $OTU_{0.97}$	Chao1	inverse Simpson	SSOabs [%]	SSOrel [%]	DSOabs [%]	Faith's PD
SSG01	45,980	5,446	9,231	69	1.5	48.1	8.1	295
SSG02	46,555	5,032	7,977	87	1.8	41.6	7.5	284
SSG03	53,143	5,235	8,673	85	2.3	44.7	7.6	297
SSG04	55,507	3,426	5,260	68	2.2	37.7	9.0	222
SSG05	58,205	5,007	8,764	114	2.1	47.2	7.5	280
SSG06	53,230	4,373	7,070	112	2.1	43.3	8.7	253
SSG07	56,972	4,369	7,327	103	1.8	44.8	6.4	253
SSG08	55,758	4,088	7,716	49	1.9	52.3	7.7	236
SSG09	52,930	5,470	9,742	128	1.9	48.2	7.9	292
SSG10	47,116	5,198	8,888	63	1.3	48.5	7.9	277
SSG11	50,657	4,407	7,180	92	2.2	42.6	8.2	253
SSG12	58,769	4,126	7,293	44	1.9	49.8	8.4	227
SSG13	46,215	5,359	9,787	82	1.8	49.9	7.0	288
SSG14	44,901	5,160	9,008	136	1.8	46.2	7.4	290
SSG15	47,901	4,866	8,783	78	1.8	50.4	7.6	265
SSG16	46,828	5,955	9,949	106	1.9	44.0	7.6	326
SSG17	45,131	6,031	10,692	58	1.8	50.8	9.1	317
bulk1	75,134	6,759	13,059	230	3.9	51.2	10.0	348
bulk2	129,394	6,797	13,119	215	4.0	51.4	10.8	354
bulk3	137,585	6,924	14,155	226	4.2	52.3	9.8	358

Table S3: Beta-diversity.

Genetic similarity between single sand grain and bulk sediment communities measured by UniFrac and expressed as shared phylogenetic branch length. Color code corresponds to proportion of shared branch length: low (red) to high proportion (green). Panel A. Unweighted UniFrac, B. Weighted UniFrac. Calculcations performed on $OTU_{0.97}$ representative sequences of subsampled data sets (N=44.901).

A

	SSG 1	SSG 2	SSG 3	SSG 4	SSG 5	SSG 6	SSG 7	SSG 8	SSG 9	SSG 10	SSG 11	SSG 12	SSG 13	SSG 14	SSG 15	SSG 16	SSG 17	Bulk 1	Bulk 2
SSG2	46																		
SSG3	47	49																	
SSG4	42	44	44																
SSG5	47	46	48	43															
SSG6	44	41	44	42	47														
SSG7	46	46	49	45	47	46													
SSG8	42	40	43	40	42	42	45												
SSG9	48	48	48	43	49	44	47	42											
SSG10	44	46	47	42	46	43	48	46	45										
SSG11	45	44	47	43	47	44	48	42	47	45									
SSG12	43	43	44	41	42	42	45	44	44	43	43								
SSG13	46	48	48	43	48	45	49	45	49	48	46	43							
SSG14	49	46	48	42	44	42	47	43	46	46	44	43	46						
SSG15	44	44	46	41	46	44	47	48	46	48	44	43	49	45					
SSG16	50	49	49	42	46	41	46	39	47	45	44	40	46	49	43				
SSG17	46	47	47	41	44	41	44	40	45	46	42	40	45	48	43	50			
Bulk1	44	45	46	40	44	41	44	40	45	44	43	40	46	43	43	46	45		
Bulk2	44	45	46	41	44	41	44	40	46	44	42	41	45	43	43	46	46	54	
Bulk3	45	45	46	40	45	41	44	39	45	44	42	40	45	44	43	46	45	54	55

	SSG to SSG	Bulk to bulk	SSG to bulk
Min	39	54	39
Max	50	55	46
Mean	45	54	44

B

	SSG 1	SSG 2	SSG 3	SSG 4	SSG 5	SSG 6	SSG 7	SSG 8	SSG 9	SSG 10	SSG 11	SSG 12	SSG 13	SSG 14	SSG 15	SSG 16	SSG 17	Bulk 1	Bulk 2
SSG2	70																		
SSG3	76	78																	
SSG4	68	69	72																
SSG5	78	71	82	68															
SSG6	75	71	78	62	82														
SSG7	71	75	81	63	77	77													
SSG8	55	62	64	50	60	63	72												
SSG9	80	75	82	69	81	76	79	61											
SSG10	71	72	84	66	81	78	82	66	79										
SSG11	69	71	75	58	71	71	75	65	76	71									
SSG12	54	61	61	50	56	59	63	80	58	60	63								
SSG13	70	72	75	60	75	74	81	75	78	78	76	69							
SSG14	70	76	75	60	70	71	85	72	76	75	77	65	79						
SSG15	67	70	76	58	73	76	83	74	75	79	72	64	81	79					
SSG16	75	78	78	68	74	72	75	60	80	73	78	59	74	79	70				
SSG17	66	67	74	66	74	70	71	59	69	77	61	53	66	67	69	68			
Bulk1	71	65	71	59	72	72	74	61	73	73	70	54	70	71	71	74	73		
Bulk2	71	68	73	60	72	73	76	62	74	74	73	56	72	74	72	76	71	93	
Bulk3	71	68	73	60	72	74	76	63	74	74	74	57	72	74	73	76	71	93	96

	SSG to SSG	Bulk to bulk	SSG to bulk
Min	50	93	54
Max	85	96	76
Mean	71	94	70

Table S4: Fraction of bulk sediment $OTU_{0.97}$ **present on a single sand grain.** The calculations were performed with different subsets of $OTU_{0.97}$: i) all $OTU_{0.97}$, ii) $OTU_{0.97}$ contributing > 0.01% to total sequences (excluding rare biosphere according to Galand and colleagues (2009), and iii) $OTU_{0.97}$ contributing > 0.1% to total sequences (excluding rare biosphere according to Pedrós-Alió (2012). Data from bulk sediment samples (bulk_1 to bulk_3) were pooled prior to analysis.

	**$OTU_{0.97}$ no.**	**SSG 1**	**SSG 2**	**SSG 3**	**SSG 4**	**SSG 5**	**SSG 6**	**SSG 7**	**SSG 8**	**SSG 9**	**SSG 10**	**SSG 11**	**SSG 12**	**SSG 13**	**SSG 14**	**SSG 15**	**SSG 16**	**SSG 17**
$OTU_{0.97}$ *	22,505	37	37	39	27	36	31	34	28	39	36	33	30	38	35	35	42	40
$OTU_{0.97}$ (>0.1‰)**	5,173	63	64	66	48	61	53	61	48	67	61	57	52	65	60	58	69	63
$OTU_{0.97}$ (>1‰)***	803	87	85	85	73	86	80	84	77	89	84	82	77	88	83	82	87	85

*: Each sample subsampled to 44,901 reads; **: Each sample subsampled to 40,449 reads; ***: Each sample subsampled to 29,378 reads

Supplementary References

Acinas SG, Ferrera I, Sarmento H, Díez-Vives C, Forn I, Ruiz-González C, et al. (2015). Validation of a new catalysed reporter deposition-fluorescence in situ hybridization probe for the accurate quantification of marine *Bacteroidetes* populations. Environ Microbiol 17: 3557-3569.

Allen HK, Bayles DO, Looft T, Trachsel J, Bass BE, Alt DP, et al. (2016). Pipeline for amplifying and analyzing amplicons of the V1–V3 region of the 16S rRNA gene. BMC Research Notes 9: 380.

Amann RI, Binder BJ, Olson RJ, Chisholm SW, Devereux R, Stahl DA. (1990). Combination of 16S rRNA-targeted oligonucleotide probes with flow cytometry for analyzing mixed microbial populations. Appl Environ Microbiol 56: 1919-1925.

Bray JR, and Curtis JT. (1957). An ordination of the upland forest communities of southern Wisconsin. Ecol Monogr 27: 326-349.

Chao A. (1984). Nonparametric estimation of the number of classes in a population. Scand J Stat 11: 265-270.

Chen J. (2012). GUniFrac: Generalized UniFrac distances. R-package version 1.0.

Daims H, Brühl A, Amann R, Schleifer KH, Wagner M. (1999). The domain-specific probe EUB338 is insufficient for the detection of all *Bacteria*: Development and evaluation of a more comprehensive probe set. Syst Appl Microbiol 22: 434-444.

Daims H, Nielsen JL, Nielsen PH, Schleifer KH, Wagner M. (2001). *In situ* characterization of *Nitrospira*-like nitrite-oxidizing bacteria active in wastewater treatment plants. Appl Environ Microbiol 67: 5273-5284.

Daims H, Lebedeva EV, Pjevac P, Han P, Herbold C, Albertsen M, et al. (2015). Complete nitrification by *Nitrospira* bacteria. Nature 528: 504-509.

Dyksma S, Bischof K, Fuchs BM, Hoffmann K, Meier D, Meyerdierks A, et al. (2016). Ubiquitous *Gammaproteobacteria* dominate dark carbon fixation in coastal sediments. ISME J 10: 1939-1953.

Edgar RC, Haas BJ, Clemente JC, Quince C, Knight R. (2011). UCHIME improves sensitivity and speed of chimera detection. Bioinformatics 27: 2194-2200.

Eickhorst T, Tippkötter R. (2008). Detection of microorganisms in undisturbed soil by combining fluorescence in situ hybridization (FISH) and micropedological methods. Soil Biol Biochem 40: 1284-1293.

Faith DP. (1992). Conservation evaluation and phylogenetic diversity. Biol Conserv 61: 1-10.

Galand PE, Casamayor EO, Kirchman DL, and Lovejoy C. (2009). Ecology of the rare microbial biosphere of the Arctic Ocean. Proc Natl Acad Sci USA 106: 22427-22432.

Gower JC (1975). Generalized Procrustes analysis. Psychometrika 40:33-51

Houston AN, Otten W, Baveye PC, Hapca S. (2013). Adaptive-window indicator kriging: A thresholding method for computed tomography images of porous media. Comput Geosci-Uk 54: 239-248.

Kembel SW, Cowan PD, Helmus MR, Cornwell WK, Morlon H, Ackerly DD, et al. (2010). Picante: R tools for integrating phylogenies and ecology. Bioinformatics 26: 1463-1464.

Kozich JJ, Westcott SL, Baxter NT, Highlander, SK, Schloss PD. (2013). Development of a dual-index sequencing strategy and curation pipeline for analyzing amplicon sequence data on the MiSeq Illumina sequencing platform. Appl Environ Microbiol 79: 5112-5120.

Lozupone C, and Knight R. (2005). UniFrac: a new phylogenetic method for comparing microbial communities. Appl Environ Microbiol 71: 8228-8235.

Ludwig W, Strunk O, Westram R, Richter L, Meier H, Yadhukumar, et al. (2004). ARB: a software environment for sequence data. Nucleic Acids Res 32: 1363–1371.

Manz W, Amann R, Ludwig W, Wagner M, Schleifer K-H. (1992). Phylogenetic oligodeoxynucleotide probes for the major subclasses of *Proteobacteria*: problems and solutions. Syst Appl Microbiol 15: 593-600.

Neef A, Amann R, Schlesner H, Schleifer K-H. (1998). Monitoring a widespread bacterial group: in situ detection of *Planctomycetes* with 16S rRNA-targeted probes. Microbiol 144: 3257-3266.

Oksanen J, Guillaume Blanchet F, Kindt R, Legendre P, Minchin PR, O'Hara RB, et al. (2013). vegan: Community ecology package. R package version 2.0-10.

Paradis E, Claude J, and Strimmer K. (2004). APE: analyses of phylogenetics and evolution in R language. Bioinformatics 20: 289-290.

Pedrós-Alió C. (2012). The rare bacterial biosphere. Annu Rev Mar Sci 4: 449-466.

Price MN, Dehal PS, and Arkin AP. (2010). FastTree 2 - approximately maximum-likelihood trees for large alignments. PloS One 5: e9490.

Pruesse E, Quast C, Knittel K, Fuchs BM, Ludwig WG, Peplies J, and Glöckner FO. (2007). SILVA: a comprehensive online resource for quality checked and aligned ribosomal RNA sequence data compatible with ARB. Nucleic Acids Res 35: 7188-7196.

Probandt D, Knittel K, Tegetmeyer HE, Ahmerkamp S, Holtappels M, Amann R. (2017). Permeability shapes bacterial communities in sublittoral surface sediments. Environ Microbiol 19: 1584-1599.

Pruesse E, Quast C, Knittel K, Fuchs, BM, Ludwig W, and Peplies J. (2007). SILVA: a comprehensive online resource for quality checked and aligned ribosomal RNA sequence data compatible with ARB. Nucleic Acids Res 35: 7188–7196.

Quast C, Pruesse E, Yilmaz P, Gerken J, Schweer T, Yarza P, et al. (2013). The SILVA ribosomal RNA gene database project: improved data processing and web-based tools. Nucleic Acids Res 41: D590-D596.

Schloss PD, Westcott SL, Ryabin T, Hall JR, Hartmann M, Hollister EB. et al. (2009). Introducing mothur: open-Source, platform-independent, community-supported software for describing and comparing microbial communities. Appl Environ Microbiol 75: 7537-7541.

Simpson EH. (1949). Measurement of diversity. Nature 163: 688-688.

Siyambalapitiya N, Blackall LL. (2005). Discrepancies in the widely applied GAM42a fluorescence in situ hybridisation probe for *Gammaproteobacteria*. FEMS Microbiol Lett 242: 367-373.

Stahl DA, Amann R. (1991). Nucleic acid techniques in bacterial systematics. in: Stackebrandt. E.. Goodfellow. M. (Eds.). John Wiley & Sons Ltd., Chichester. England. pp. 205-248.

Teira E, Reinthaler T, Pernthaler A, Pernthaler J, Herndl GJ. (2004). Combining catalyzed reporter deposition-fluorescence in situ hybridization and microautoradiography to detect substrate utilization by bacteria and archaea in the deep ocean. Appl Environ Microbiol 70: 4411-4414.

van Kessel MAHJ, Speth DR, Albertsen M, Nielsen PH, Op den Camp HJM, Kartal B, et al. (2015). Complete nitrification by a single microorganism. Nature 528: 555-559.

Wallner G, Amann R, Beisker W. (1993). Optimizing fluorescent in situ hybridization with rRNA-targeted oligonucleotide probes for flow cytometric identification of microorganisms. Cytometry 14: 136-143.

Westcott SL, Schloss PD. (2017). OptiClust, an improved method for assigning amplicon-based sequence data to operational taxonomic units. mSphere 2: 10.1128/mSphereDirect.00073-00017.

Yarza P, Yilmaz P, Pruesse E, Glöckner FO, Ludwig W, Schleifer K-H, et al. (2014). Uniting the classification of cultured and uncultured bacteria and archaea using 16S rRNA gene sequences. Nat Rev Microbiol 12: 635-645.
